# Sex-Related Differences in Lactotroph Tumor Aggressiveness Are Associated With a Specific Gene-Expression Signature and Genome Instability

**DOI:** 10.3389/fendo.2018.00706

**Published:** 2018-11-30

**Authors:** Anne Wierinckx, Etienne Delgrange, Philippe Bertolino, Patrick François, Philippe Chanson, Emmanuel Jouanneau, Joël Lachuer, Jacqueline Trouillas, Gérald Raverot

**Affiliations:** ^1^Institut Universitaire de Technologie, Université Lyon 1, Université de Lyon, Lyon, France; ^2^Centre de Recherche en Cancérologie de Lyon (CRCL), INSERM U1052, CNRS UMR5286, Université de Lyon, Lyon, France; ^3^ProfileXpert, SFR-Est, CNRS UMR-S3453, INSERM US7, Lyon, France; ^4^Service d'Endocrinologie, CHU UCL Namur, Université catholique de Louvain, Ottignies-Louvain-la-Neuve, Belgium; ^5^Service de Neurochirurgie, CHU de Tours, Tours, France; ^6^Service d'Endocrinologie et des Maladies de la Reproduction, Assistance Publique-Hôpitaux de Paris, Centre de Référence des Maladies Rares de l'Hypophyse, Hôpital Bicêtre, Le Kremlin-Bicêtre, France; ^7^Faculté de Médecine Paris-Sud, UMR S-1185, Université Paris-Sud, Université Paris-Saclay, Le Kremlin-Bicêtre, France; ^8^Service de Neurochirurgie Groupement Hospitalier Est, Hospices Civils de Lyon, Bron, France; ^9^Faculté de Médecine Lyon-Est, Université Lyon 1, Université de Lyon, Lyon, France; ^10^Département d'Endocrinologie, Centre de Référence pour les Maladies Hypophysaires Rares (HYPO), Groupement Hospitalier EST, Hospices Civils de Lyon, Université de Lyon, Lyon, France

**Keywords:** pituitary tumors, gene expression, estrogen signaling, sexual dimorphism, chromosome, aggressiveness

## Abstract

Sex-related differences have been reported in various cancers, in particular men with lactotroph tumors have a worse prognosis than women. While the underlying mechanism of this sexual dimorphism remains unclear, it has been suggested that a lower estrogen receptor alpha expression may drive the sex differences observed in aggressive and malignant lactotroph tumors that are resistant to dopamine agonists. Based on this observation, we aimed to explore the molecular importance of the estrogen pathway through a detailed analysis of the transcriptomic profile of lactotroph tumors from 20 men and 10 women. We undertook gene expression analysis of the selected lactotroph tumors following their pathological grading using the five-tiered classification. Chromosomic alterations were further determined in 13 tumors. Functional analysis showed that there were differences between tumors from men and women in gene signatures associated with cell morphology, cell growth, cell proliferation, development, and cell movement. Hundred-forty genes showed an increased or decreased expression with a minimum 2-fold change. A large subset of those genes belonged to the estrogen receptor signaling pathway, therefore confirming the potent role of this pathway in lactotroph tumor sex-associated aggressiveness. Genes belonging to the X chromosome, such as *CTAG2, FGF13*, and *VEGF-D*, were identified as appealing candidates with a sex-linked dysregulation in lactotroph tumors. Through our comparative genomic hybridization analyses (CGH), chromosomic gain, in particular chromosome 19p, was found only in tumors from men, while deletion of chromosome 11 was sex-independent, as it was found in most (5/6) of the aggressive and malignant tumors. Comparison of transcriptomic and CGH analysis revealed four genes (*CRB3, FAM138F, MATK, and STAP2*) located on gained regions of chromosome 19 and upregulated in lactotroph tumors from men. MATK and STAP2 are both implicated in cell growth and are reported to be associated with the estrogen signaling pathway. Our work confirms the proposed involvement of the estrogen signaling pathway in favoring the increased aggressiveness of lactotroph tumors in men. More importantly, we highlight a number of ER-related candidate genes and further identify a series of target molecules with sex-specific expression that could contribute to the aggressive behavior of lactotroph tumors in men.

## Introduction

Epidemiological data indicates that tumors such as lung cancer, hepatocarcinoma, and melanoma have a worse prognosis in men than in women. This observation is also true for both metastatic and primary brain tumors including gliomas, meningiomas, and a subset of pituitary tumors that produce prolactin. These latter tumors, defined as pituitary lactotroph tumors, are larger in men than in women (1), less sensitive to dopamine agonists (2), and their proliferative activity is reported to be higher in men and older women (>40 years of age) than in young women (3). The longer diagnostic delay in men cannot solely explain the sex-related differences in lactotroph tumors. Indeed, in a large surgical series of patients with lactotroph tumors, we previously demonstrated the increased aggressiveness of this type of tumor related to a higher proliferative index among men (2). More recently, we confirmed the sexual dimorphism that exists in lactotroph pituitary tumors by demonstrating that low expression of estrogen receptor alpha (ERα) is more frequently observed in men and further associated in both sexes with high-grade lactotroph tumors that are resistant to therapeutic treatments (4). Besides the sex specificity that exists in terms of hormone regulation and secretion, the most evident differences between men and women lie in their epigenome and the existence of X and Y sex chromosomes. Among X located genes, the androgen receptor (AR), glucose metabolic enzymes, proteins of the apoptotic cascade are expressed in normal tissues and modified in various tumors. Moreover, other X-located genes as cancer-testis antigens are expressed in numerous tumors, while in normal tissues, the expression of most of them remains restricted within the testis and the placenta (5). Expression of cancer-testis antigens is regulated by epigenetic mechanisms and could be associated with tumor progression (6). Increasing data also support the role of genes located on the Y chromosome, such as the candidate tumor suppressor *TMSB4Y* (7), a hypothesis further confirmed by the loss of the Y chromosome that is observed in cancers (8, 9). Surprisingly, little is known about the molecular mechanisms that drives the sexual dimorphism observed in pituitary lactotroph tumors, and studies comparing gene expression between tumors in men and women are lacking.

Here, we addressed these questions in order to delineate the mechanisms and identify genes that drive the sex specificity that exists in aggressive lactotroph tumors. While our data confirm the implication of estrogen signaling in the sexual dimorphism observed in these tumors, it further highlights a number of candidate genes and pathways that could represent appealing targets contributing to sex-related differences in lactotroph tumors.

## Materials and methods

### Human pituitary tumors

Thirty frozen tumors stored at the Neurobiotec Bank (Lyon, France) were selected from a series of 89 lactotroph pituitary tumors we had previously used to report the existence of ERα-associated sex-related differences among men and women (4). All included tumors were resected between 1989 and 2005 by the transsphenoidal route and were further shown to be positive for prolactin expression by immunohistochemistry. Their grading was carried out according to the clinicopathological classification we have previously established (10). Briefly, they were classified into five grades (grades 1a, 1b, 2a for non-aggressive tumors, grade 2b for aggressive, and grade 3 for malignant tumors). The expression of ERα in those tumors was quantified as previously reported (4). The surrounding normal pituitary of each non-invasive microadenoma was macroscopically discarded by manual dissection to avoid any potent contamination that could interfere with our gene expression analysis. A subsequent qRT-PCR was also performed on the 30 selected tumors to address prolactin (*PRL*), growth hormone (*GH*), proopiomelanocortin (*POMC*), and luteinizing hormone *LH*β in order to exclude PRL/GH co-producing tumors and normal tissues that co-express POMC/LHβ. Note that although normal pituitary tissues are not representative of normal lactotroph cells, we used a pool of normal pituitary from men and women as control references (data not shown). Microarray data for transcriptomic analysis were obtained from patients participating to the HYPOPRONOS (Programme Hospitalier de Recherche Clinique National 27-43) study, and genotyping and copy number alteration (CNA) analysis from patients included in the PITUIGENE study PHRC-INCa 2012 (ClinicalTrials.gov Identifier: NCT01903967). These studies were approved by the ethics committee of Lyon, and informed consent was obtained from each patient according to French law.

### Transcriptomic analysis

Total RNA was extracted from pituitary tumors using Trizol (Invitrogen), according to the manufacturer's protocol (Invitrogen, Carlsbad, California, USA). For qRT-PCR, total RNA was subjected to DNAse treatment using an RNeasy minikit (Qiagen, Hilden, Germany) according to the manufacturer's protocol. Total RNA yield was measured by the OD260, the purity was checked by a A260/A280 ratio of 1.9-2.1, and the quality was evaluated on nanochips with the Agilent 2100 Bioanalyzer (Agilent Technologies, Palo Alto, CA, USA) according to the manufacturer's protocol.

### RNA amplification

Total RNA (2 μg) was amplified and biotin-labeled by a round of *in vitro* transcription with a Message Amp aRNA kit (Ambion, Austin, Texas, USA) following the manufacturer's protocol. Before amplification, spikes of synthetic mRNA at different concentrations were added to all samples; these positive controls were used to ascertain the quality of the process. aRNA yield was measured using a UV spectrophotometer and the quality on nanochips with the Agilent 2100 Bioanalyzer (Agilent).

### Array hybridization and processing

Ten micrograms of biotin-labeled aRNA was fragmented using 5 μl of fragmentation buffer in a final volume of 20 μl and was then mixed with 240 μl of Amersham hybridization solution (GE Healthcare Europe GmbH, Freiburg, Germany) and injected onto CodeLink Uniset Human Whole Genome bioarrays containing 5,5000 human oligonucleotide gene probes (GE Healthcare Europe GmbH, Freiburg, Germany) as described previously (11). Arrays were hybridized overnight at 37°C at 300 rpm in an incubator. The slides were washed in stringent TNT buffer at 46°C for 1 h, then a streptavidin-cy5 (GE Healthcare) detection step was performed. Each slide was incubated for 30 min in 3.4 ml of streptavidin-cy5 solution, was then washed four times in 240 ml of TNT buffer, rinsed twice in 240 ml of water containing 0.2%Triton X-100, and dried by centrifugation at 600 rpm. The slides were scanned using a Genepix 4000B scanner (Axon, Union City, USA) and Genepix software, with the laser set at 635 mm, the laser power at 100%, and the photomultiplier tube voltage at 60%. The scanned image files were analyzed using CodeLink expression software, version 4.0 (GE Healthcare), which produces both a raw and normalized hybridization signal for each spot on the array. Transcriptomic data have been deposited in Gene Expression Omnibus under the accession number GSE120350.

### Microarray data analysis

The relative intensity of the raw hybridization signal on arrays varies in different experiments. CodeLink software was therefore used to normalize the raw hybridization signal on each array to the median of the array (median intensity is 1 after normalization) for better cross-array comparison. The threshold of detection was calculated using the normalized signal intensity of the 100 negative control samples in the array; spots with signal intensities below this threshold are referred to as “absent.” Quality of processing was evaluated by generating scatter plots of positive signal distribution. Signal intensities were then converted to log base 2 values. Differential expression analysis was performed using RStudio (http://www.rstudio.org) to isolate differentially expressed mRNAs between men and women lactotroph tumors. A mRNA transcript was considered differentially expressed if the difference gave a *p* ≤ 0.05 with the Student's *t*-test, and showed a minimal 2-fold variation. Functional Analysis were created with Ingenuity Pathway Analysis software (IPA®, QIAGEN Redwood City, www.qiagen.com/ingenuity).

### Quantitative gene expression analysis through qRT-PCR

Total RNA (0.5 μg) was reverse transcribed using MMLV reverse transcriptase (Invitrogen). The absence of contaminating genomic DNA in the RT reactions was checked by qRT-PCR directly on total RNA. The cDNA synthesized was measured using qRT-PCR (SYBR Green PCR, LightCycler, Roche Diagnostics Indianapolis, USA) following manufacturer's recommendations. The LightCycler experimental run consisted of an initial Taq activation at 95°C for 10 min and 45 cycles of the amplification and quantification program (95°C for 15 s, 60°C for 5 s, and 72°C for 10 s, with a single fluorescence measurement). The specificity of PCR amplification was always analyzed with a melting curve program (69–95°C) with a heating rate of 0.1°C per second and continuous fluorescence measurement. Primers were designed using Primer3 software (Whitehead Institute/MIT, USA) to insure their respective Tm were between 59 and 61°C and their use produces amplicons between 100 and 150 bp.

### Comparative genomic hybridization (CGH) analysis

Genomic DNA was isolated from 13 tumoral and 1 pool of normal pituitary frozen fragments using the QIAamp DNA micro kit (Qiagen, Hilden, Germany), quantified with nanodrop (NanoDrop, Wilmington, DE, USA), and quality verified on agarose gel. Genotyping and CNA analysis was performed using the Affymetrix Genome-wide human SNP array 6.0 chip following manufacturer protocol (Affymetrix, Santa Clara, CA, USA). Briefly, 250 ng of genomic DNA was digested by the nsp/sty enzyme, adaptor ligated and PCR amplified using a single primer with titanium Taq polymerase (Invitrogen, Carlsbad, California, USA). Amplified PCR products were pooled, concentrated, and fragmented with DNase I. Products were subsequently labeled, denatured, and hybridized overnight to the respective arrays. Arrays were washed using the Affymetrix 450 fluidic array station and scanned using the GeneChip scanner 3000 7G. We generated CEL files using the Affymetrix GeneChip Command Console software (AGCC) 3.0. The tissues and reference 103 genomic DNA were processed in a same batch and hybridized using the ProfileXpert platform. CGH and genotyping data have been deposited in Gene Expression Omnibus under the accession number GSE 22615.

### Copy number alterations (CNA) analysis

Affymetrix CEL files were extracted using the Genotyping Console software version 3.0 (Affymetrix). For SNP genotyping, we used the Birdseed (v2.2) analysis algorithm. Accuracy of genotyping was checked by performing a concordance test between the processed reference 103 and a pre-processed reference 103 (Affymetrix). The test of concordance showed a 99.79% homology between the two genotypes indicating a good performance of the platform. Moreover, samples showing a call rate >96% and a median of the absolute values of all pairwise differences (MAPD) metric <4 were considered in further analysis. CNA analysis was performed using Partek Genomics Suite version 6.4 (Partek, St Louis, MO) following normalization by invariant set normalization procedure and computed signal intensities using perfect match and mismatch (PM/MM) model-based expression. Raw copy number data was computed using a batch of 270 normal external controls samples from the International HapMap project and used as reference. To remove alterations not associated with tumor phenotype, copy number variation was also analyzed on a pool of normal pituitary samples processed simultaneously in the same batch as tumor samples and compared to the references. Inferred copy numbers were predicted using genomic segmentation algorithm. Only copy number alterations with cut-offs of >2.7 copies for gain and <1.3 for loss were considered.

## Results

### Clinical and pathological features of the analyzed cohort of lactotroph tumors

In order to explore the genes and mechanisms related to estrogen signaling in the sex-associated aggressiveness of lactotroph tumors, we selected a cohort of 30 tumors from 20 male and 10 female patients. Tumors were classified into five grades, ranging from benign (grades 1a-1b), invasive (grade 2a), suspected of malignancy (grade 2b), to malignant with metastasis (grade 3). Detailed clinical features of the selected tumors are summarized in Table 1. Interestingly, while the number of patients is rather limited, with an overrepresentation of tumors from men, the sex-related clinicopathological differences previously reported are obvious (4). Tumors in men were significantly larger, mostly invasive, and negative for ERα. Proliferation markers and tumor grades were also higher in men although this was not statistically significant. Taken together, these observations indicate that our cohort of 30 lactotroph tumors present consistent sex-related differences that should facilitate the identification of sex-associated candidate genes.

**Table 1:** Sex-related comparison of clinical, biological, and pathological characteristics in 30 patients with lactotroph tumors.

	**Women (*n* = 10)**	**Men (*n* = 20)**
**Age (years)**	35 ± 3	51 ± 2
**MRI DATA**		
• Tumor size, (mm)	11 ± 1	27 ± 3
- <10 mm, *n*	3	1
−10–40 mm, *n*	7	13
->40mm, *n*	0	6
• Invasive tumors, *n* (%)	3 (30)	15 (75)
**TUMOR CHARACTERISTICS**		
•ERα expression (IR score)	7 ± 1	3 ± 1
•**Proliferative markers**		
-Mitotic count	1 ± 1	4 ± 1
-Ki-67 (%)	0.8 ± 0.5	2.5 ± 0.7
-p53 (%)	0.3 ± 0.1	0.6 ± 0.2
•**Prognostic classification**		
-Grade 1a, *n*	6	4
-Grade 1b, *n*	1	1
-Grade 2a, *n*	1	8
-Grade 2b (–>3^*^), *n*	2	7 (2)

* *Two of the seven male tumors were classified grade 3 based on metastasis during the follow-up. For continuous variables, results are presented as the mean ± SE (median)*.

### Transcriptomic analyses reveal sex-specific gene expression differences between lactotroph tumors from men and women

Having validated the sexual dimorphism of our cohort, we next performed a gene expression analysis. Transcriptomic profiling was achieved through the use of a CodeLink Uniset Human Whole Genome Bioarray. Comparative exploration of male and female expression profiles revealed that 140 genes showed a significant deregulation of at least 2-fold between lactotroph tumors from men and women (Table S1), with an overrepresentation of genes with increased expression. Indeed, while 120 genes were increased, only 20 showed a reduced expression. Interestingly, we found that nearly 10% (11/120) of the genes showing an increased expression in men were located on the Y chromosome (*DDX3Y, EIF1AY, KDM5D, NLGN4Y, PRKY, RPS4Y1, RPS4Y2, TTTY14, TXLNGY, USP9Y, ZFY*). Surprisingly, analysis of the Y chromosome-located *TMSB4Y* tumor suppressor did not reveal any altered expression between lactotroph tumors from men and women. Similarly, our analysis revealed that almost 6% (7/120) of the genes overexpressed in male tumors were located on the X chromosome (*FGF13, VEGFD, CTAG2, SLC6A8, DDX3P1, FRMPD4, TMEM35A*). Out of these candidates, we found that *FGF13* was already expressed at higher levels in normal male pituitaries compared to female ones, whereas *VEGFD, CTAG2*, and *SLC6A8* showed comparable expression between the sexes in normal pituitary tissues. Interestingly, *FGF13* and *VEGFD* are both involved in mechanisms such as angiogenesis, cell growth/proliferation, and control of cellular movement/morphology. *VEGFD* is further involved in cell cycle control (12, 13), supporting the overall importance of this candidate in the sex-linked aggressiveness of lactotroph tumors. Besides *FGF13* and *VEGFD*, the candidate *CTAG2* is involved in cellular movement and has previously been associated with invasion in breast cancer (6). As shown in Table 2, correlative analysis revealed that, in men, *CTAG2* expression was strongly correlated with known lactotroph tumor aggressiveness markers implicated in the cell cycle (*CENPE, AURKB, CCNB1, ADAMTS6*) (11). Finally, it is interesting to note that the overexpression of the phosphocreatine transporter gene *SLC6A8* suggests the existence of metabolic advantages in male tumors.

**Table 2:** Correlation between CTAG2 and markers of aggressiveness in lactotroph tumors.

**Genes**	**Major functions**	**Tumors in women**		**Tumors in men**	
		**Pearson correlation**	***p*-value**	**Pearson correlation**	***p*-value**
*ADAMTS6*	Development	0.32	1.9.10^−01^	0.47	1.9.10^−02^
*AURKB*	Cell cycle	0.24	2.6.10^−01^	0.93	1.2.10^−09^
*CCNB1*	Cell cycle	0.19	3.0.10^−01^	0.87	3.0.10^−07^
*CENPE*	Cell cycle	−0.02	5.2.10^−01^	0.78	2.9.10^−05^
*PTTG1*	Cell cycle	−0.15	6.6.10^−01^	0.8	1.3.10^−05^

### Chromosomic alterations in lactotroph tumors define a sex-specific gene landscape

**Figure 1:**
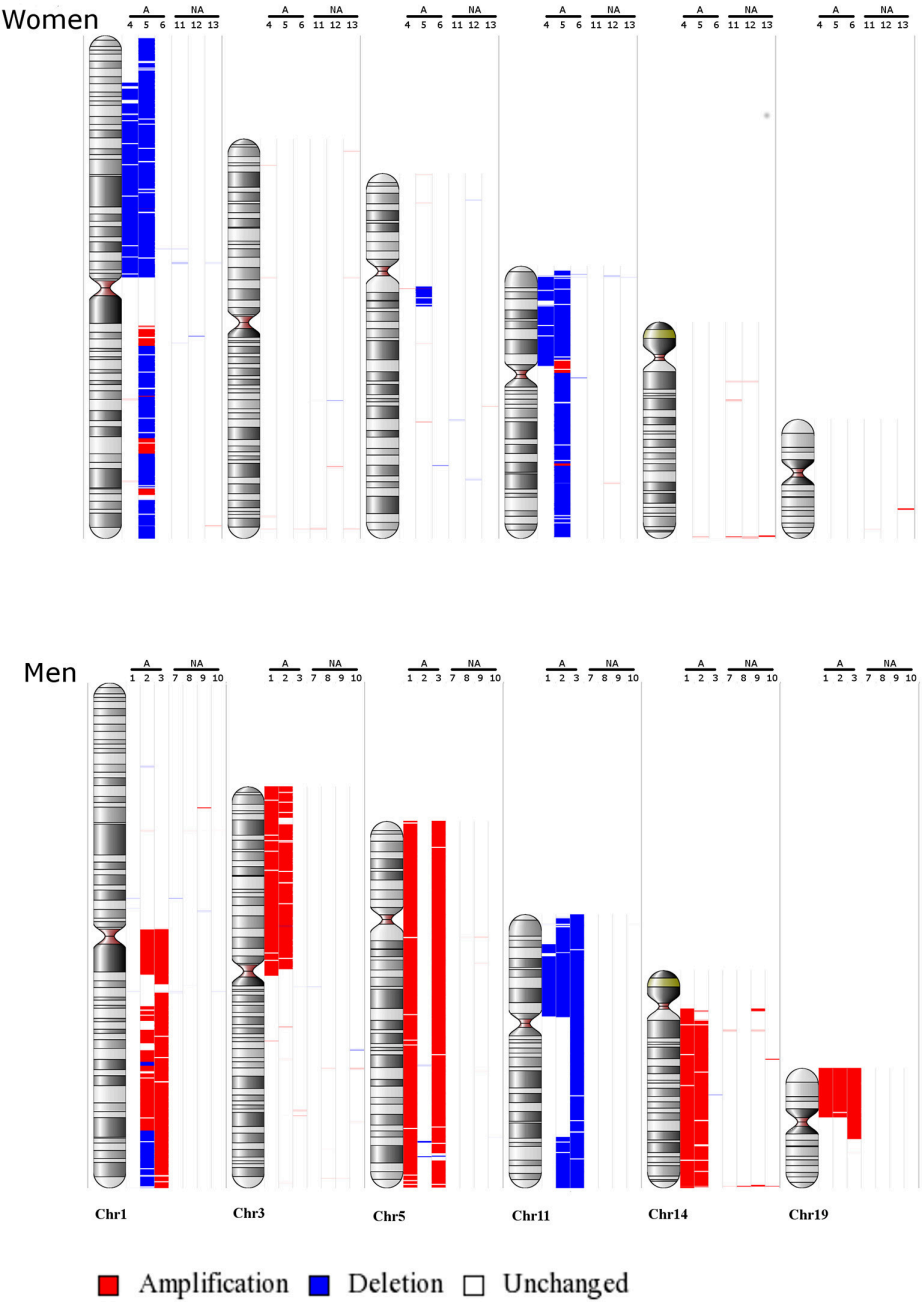
Main chromosomic abnormalities in lactotroph tumors from seven men and six women. Gains are indicated by red bars and losses by blue bars. NA, non-aggressive lactotroph tumors; A, aggressive lactotroph tumors. Genomic DNA reference was cont103 (Affymetrix).

Following the identification of a sex-specific gene expression, we further determined whether exploration of chromosomic alterations could pinpoint a genetic origin of the sexual dimorphism that exists in lactotroph tumors. Taking advantage of a CGH array we previously performed on 13 lactotroph tumors (14), of which 12 are included in the transcriptomic study, we investigated whether the observed chromosomal alterations were sex-linked. Clinical details and sex (seven men, six females) of those 13 tumors are provided in Table 3. Through this work, we confirmed the sex-independent association of several abnormalities of chromosome 1 (gain & loss) and chromosome 11 (deletion) in aggressive tumors grade 2b (10). We further found that chromosomes 3, 5, and 14 were frequently affected without any sex- or tumor grade-specific correlations. Interestingly, we observed that chromosomic abnormalities were more numerous in aggressive than in non-aggressive tumors and that a specific gain of chromosome 19p was found in three aggressive lactotroph tumors from men (Table 3 and Figure 1). Comparative analysis of CGH and transcriptomic data was subsequently carried out using the average number of known genes for each chromosome to calculate the chromosomic distribution of deregulated genes between lactotroph tumors from men and women. As summarized in Table 4, we found that chromosomes 19, 3, 2, and 5 represented the top four chromosomes with the highest percentage of deregulated genes (0.8, 0.77, 0.75, and 0.73%, respectively) that stand furthest from the median (0.53%). Having revealed chromosome 19 to be the sole chromosome subjected to sex-specific rearrangement, we wished to identify the candidate genes located within the concerned regions. We subsequently identified four genes (*CRB3, FAM138F, MATK*, and *STAP2*) that presented a minimum of a 2-fold statistically significantly increased expression in male lactotroph tumors (Table S1).

**Table 3:** Pathological and genetic data from patients with lactotroph tumors.

**Tumor number**	**Sex**	**Clinical behavior**	**Pathological group**	**Chromosome gains**	**Chromosome losses**
1	M	Recurrence, death	2b	**3p**, **5**, 8, **14q**, **19p**	**11p**
2	M	Recurrences, metastasis, death	2b->3	**1q**, **3p**, 8q, 9, **14q**, **19p**	**1q, 11**
3	M	Recurrences, metastasis, death	2b->3	**1q, 5**, 15q, **19p#**	**11**, 17p
4	F	Recurrence	2b	4q	**1p**, **11p**
5^*^	F	Recurrences, metastasis, death	2b->3	**1q**, 8q, 15q	**1**, 4, **5q**, **11**, 13q, 15, 16
6	F	Recurrence	2b	–	–
7	M	Persistence	2a	Y	–
8	M	Persistence	2a	–	15q, 2p
9	M	Remission	1a	7, 9	–
10	M	Remission	1a	8, Y	–
11	F	Remission	1a	9	–
12	F	Remission	1a	7p, 20	13q
13	F	Remission	1a	9	—

* *Not included in transcriptomic analysis. #In the publication Wierinckx et al. (14), an error occurred in case 3 of this table; it was reported to have an insertion of 19q, while the insertion is 19p as indicated here. In bold the chromosomes presented on figure 1*.

**Table 4:** Chromosomic deregulated genes between men and women in lactotroph tumors.

**Localization**	**Deregulated genes (*n*)**	**Δ Genes represented (%)**
chr19	**12**	0.80
chr3	**10**	0.77
chr2	**12**	0.75
chr5	**8**	0.73
chr13	**3**	0.60
chr9	**6**	0.60
chr8	**5**	0.59
chr16	**6**	0.59
chr7	**7**	0.58
chr1	**13**	0.57
chr11	8	0.53^*^
chr4	5	0.53^*^
chr6	6	0.44
chr14	4	0.42
chr21	1	0.33
chr18	1	0.29
chr20	2	0.29
chr17	3	0.22
chr12	2	0.17
chr22	1	0.15
chr15	1	0.13
chr10	1	0.10

* Median, in bold number of genes above the median $$\begin{array}{l} △\text{Genes represented by the chromosome }\lambda \\ \;=\left[\frac{\begin{array}{l} \text{number of genes deregulated}\\ \text{ chromosome}\lambda \end{array}}{\text{number of deregulated genes}}\right]-\left[\frac{\begin{array}{l} \text{number of genes on}\\ \text{ chromosome }\lambda \end{array}}{\text{number of total genes}}\right] \end{array}$$

### Identification of sex-associated candidate genes through a functional signature analysis

We next proceeded with gene stratification using an Ingenuity pathway analysis combined with an in-depth exploration of the scientific literature to further understand the function of the identified genes (Table S1). Using such an approach, we reviewed the 140 identified candidate genes with a focused interest in biological function relating to cancer biology, tumor progression, and aggressiveness. In this way, we found that several genes had been reported to contribute to angiogenesis (10 genes), cell growth and proliferation (29 genes), cell death and survival (6 genes), control of cell morphology (12 genes), control of cellular movement (32 genes), and development (35 genes) (Table S1). In parallel, we further categorized genes involved in normal and pathological pituitary functions and found a substantial number of the identified genes to be related to the endocrine system (10 genes), estrogen signaling (25 genes), pituitary tumors (10 genes), or to be involved in sexual dimorphism (11 genes) (Table S1). Following this classification, we cross-analyzed the two lists (*i.e* oncology processes vs. normal/pathological pituitary functions) of genes and isolated a subset of 32 candidates that were the most representative genes linked to the potent sex specificity of lactotroph tumors (Table 5).

**Table 5:** Major functions of genes deregulated by a factor of two between male and female lactotroph tumors.

**Symbol**	**Expr fold change**	**Expr *p*-value**	**Description**	**Chromosome**	**Angiogenesis**	**Cell growth and proliferation**	**Cell death and survival**	**Cell morphology**	**Cellular movement**	**Development**	**Endocrine system**	**Estrogen signaling**	**Pituitary tumors**	**Sexual dimorphism**	**Symbol**
BHLHE41	2.077	0.00365	Basic helix-loop-helix family member e41	12					x	x		x			BHLHE41
CDK8	2.599	5.59 10^−5^	Cyclin dependent kinase 8	13		x						x			CDK8
CHL1	3.117	0.0296	Cell adhesion molecule L1 like	3		x		x	x	x			x		CHL1
CTAG2	2.332	0.0242	Cancer/testis antigen 2	X					x			x			CTAG2
ENC1	2.023	0.0336	Ectodermal-neural cortex 1	5		x		x	x				x		ENC1
ERBIN	2.008	0.0191	Erbb2 interacting protein	5		x		x		x		x		x	ERBIN
ETS2	2.366	5.76 10^−4^	ETS proto-oncogene 2, transcription factor	21			x			x		x	x		ETS2
EXT1	2.181	0.00196	Exostosin glycosyltransferase 1	8		x			x	x		x			EXT1
EZR	−2.199	0.0187	Ezrin	6		x		x	x	x			x		EZR
FGF13	3.419	0.0067	Fibroblast growth factor 13	X		x	x	x	x	x		x			FGF13
FOXA1	2.35	0.0338	Forkhead box A1	14		x		x	x	x	x	x		x	FOXA1
FOXQ1	5.758	0.0393	Forkhead box Q1	6					x	x		x			FOXQ1
GADD45G	−2.656	0.0281	Growth arrest and DNA damage inducible gamma	9		x							x		GADD45G
ISL1	2.163	0.0338	ISL LIM homeobox 1	5	x	x	x		x	x	x				ISL1
KDM5D	18.032	4.12 10^−10^	Lysine demethylase 5d	Y		x								x	KDM5D
LTBP1	2.246	0.0367	Latent transforming growth factor beta binding protein 1	2	x	x	x			x		x	x	x	LTBP1
MYH7	−4.706	0.00688	Myosin heavy chain 7	14						x		x			MYH7
NMU	3.336	0.00721	Neuromedin u	4					x			x			NMU
OBSCN	2.596	0.0158	Obscurin, cytoskeletal calmodulin and titin-interacting rhogef	1			x					x			OBSCN
PITX1	2.459	0.00579	Paired like homeodomain 1	5						x	x	x	x		PITX1
PPID	2.261	0.0369	Peptidylprolyl isomerase d	4					x					x	PPID
PTGS1	2.554	0.0263	Prostaglandin-endoperoxide synthase 1	9	x					x	x	x			PTGS1
PTPRZ1	2.393	0.0496	Protein tyrosine phosphatase, receptor type z1	7	x	x		x	x	x		x			PTPRZ1
ROBO1	2.033	0.0144	Roundabout guidance receptor 1	3	x	x		x	x	x		x			ROBO1
SLC12A4	3.348	0.00534	Solute carrier family 12 member 4	16						x			x		SLC12A4
SLC2A11	2.342	0.0131	Solute carrier family 2 member 11	22		x					x		x		SLC2A11
SLC6A8	2.577	0.00231	Solute carrier family 6 member 8	X			x					x		x	SLC6A8
SNCB	2.022	0.013	Synuclein beta	5				x		x		x			SNCB
SOSTDC1	6.814	0.0103	Sclerostin domain containing 1	7						x		x			SOSTDC1
STAP2	2.122	0.00363	Signal transducing adaptor family member 2	19					x			x			STAP2
TSPAN8	3.232	0.0371	Tetraspanin 8	12					x			x			TSPAN8
VEGFD	2.27	0.0363	vascular endothelial growth factor d	X	x	x		x	x	x			x		VEGFD

### Genes involved in estrogen signaling and sexual dimorphism

**Figure 2:**
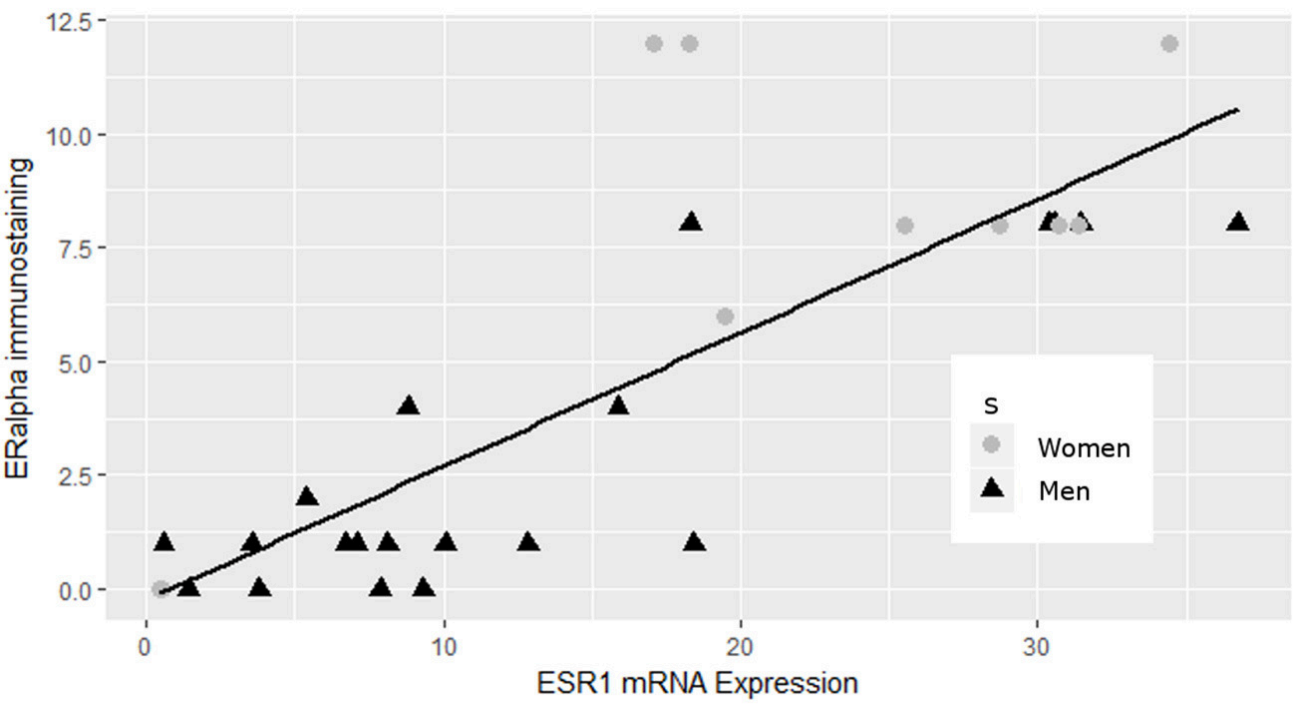
Correlation between ERα and *ESR1* mRNA expression in lactotroph tumors. ERα data from immunoblotting (4) strongly correlated (pearson correlation = 0.817, *p* = 1.767e-08) with the expression of the *ESR1* gene.

Having previously reported the contribution of the estrogen signaling pathway in the sex specificity of lactotroph tumors through the identification of a low expression level of ERα in male tumors (4), we first confirmed that the expression of the estrogen receptor 1 gene (*ESR1)* found in our analysis strongly correlated (pearson correlation = 0.817, *p* = 1.767e-08) with the protein expression of ERα addressed by immunohistochemistry in our previous work (Figure 2) (4). Despite this observation, analysis of both *ESR1* and *AR* genes, coding for ERα and the andogen receptor, respectively, did not reveal significant different expression levels between lactotroph tumors from men and women. In contrats to protein expression of ERα addressed by immunohistochemistry, expression of the *ESR1* mRNA between men and women lactotroph tumors was not significantly reduced (*FC* = −1.5; *p* = 0.13), but in the aggressive tumors in women, the level of *ESR1* mRNA is very low. Then, if we removed these two samples from our statistical analysis, we observed that *ESR1* mRNA expression was significantly lower in men (*n* = 20) than in women (*n* = 8) (*FC* = −1.9, *p* = 0.0016) lactotroph tumors. Comparing only the non-aggressive lactotroph tumors between men and women, the expression level of *ESR1* mRNA remained significantly lower in men compared to women (*FC* = −1.6; *p* = 0.031) and in normal pituitary, although not representative of the normal prolactin cells, the level of *ESR1* mRNA expression was lower in men than in women (*FC* = −2.41). Next, we assessed whether a subset of estrogen signaling-related genes could be found within our list of candidates (Table S1). Out of the 140 genes differentially expressed between male and female lactotroph tumors, we found that 25 genes related to estrogen signaling (Table S1). Out of these, 22 were also associated with oncologic processes (Table 5) and 9 were related to sex differences (Table S1 and Table 5). From these analyses, a series of appealing candidate genes were identified. Among them we found *ERBIN*, previously shown to be expressed in hepatocellular carcinoma and to promote tumorigenesis (15), and *FOXA1*, which significantly negatively correlated with *ESR1* expression (Pearson Correlation = −0.45; *p* = 0.014) and is known to regulate *ISL1* and *PPP1R14C, two* other identified estrogen-related genes. Finally, *SLC6A8* also attracted our interest based on its significant 2.6-fold overexpression in male lactotroph tumors (*p* = 0.00231) and its capacity to be inhibited by estrogens and stimulated by testosterone (16).

## Discussion

We have previously reported that lactotroph tumors that develop in men are of a higher grade and are resistant to treatment, and men have an overall worse prognosis compared to women (4). Despite this evident sexual dimorphism, there have been few studies carried out to compare gene expression between the sexes. Here, we used a comparative set of analyses involving transcriptomic and CGH experimental data obtained from lactotroph tumors from 20 men and 10 women to undertake such an analysis. We paid particular attention to the importance of the estrogen signaling pathway in sex-specific behavior due to our previous identification of a reduced ERα protein expression in male lactotroph tumors (4) and the well-established correlation between the grade of malignancy and low ERα protein expression that exists in breast tissues and bladder tumors (17–19).

**Figure 3:**
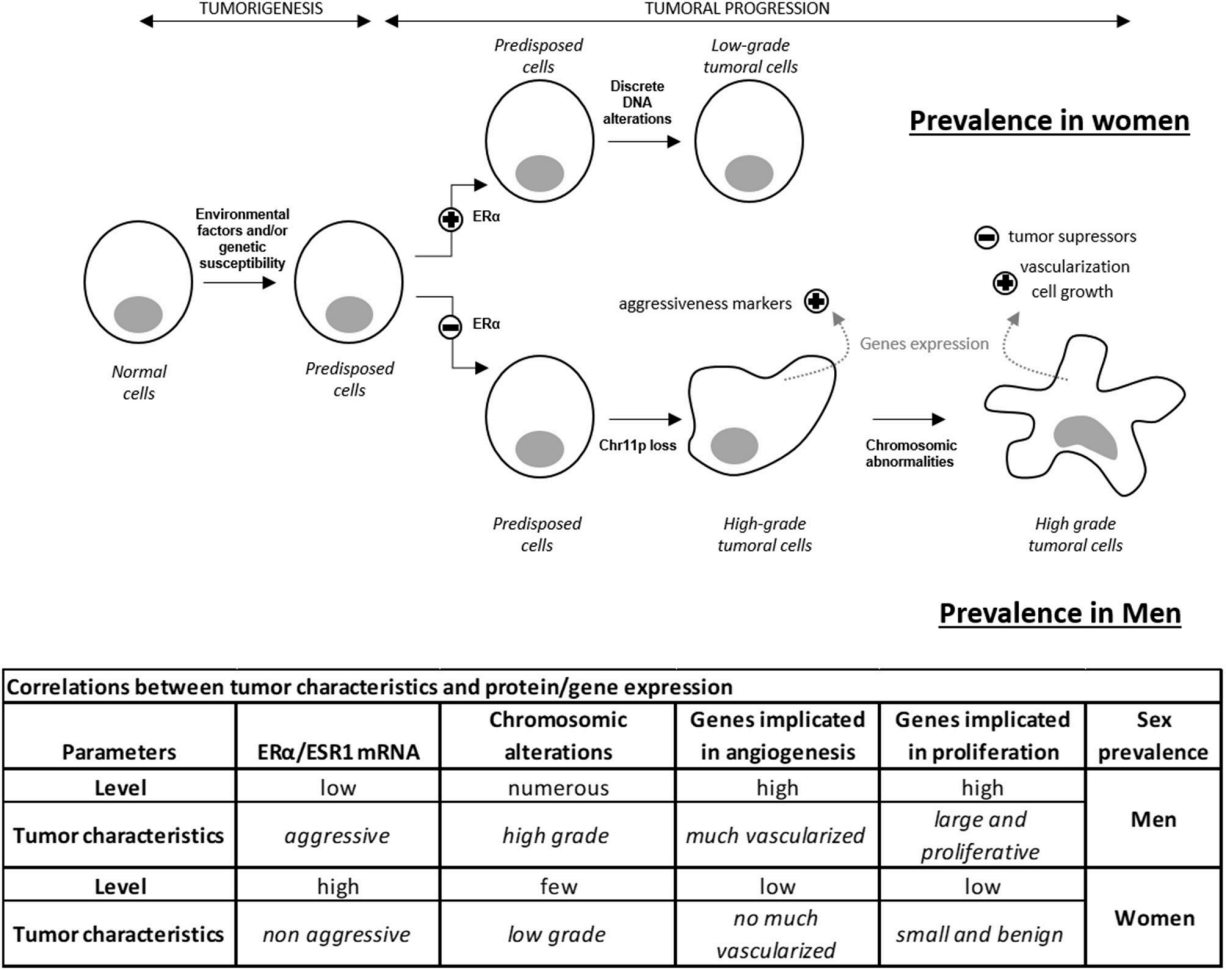
Hypothetical model of lactotroph tumor progression explaining the higher prevalence of aggressive tumors in men compared to women. ERα level influences tumor incidence and progression. A high level of ERα induces the development of lactotroph tumors and protects against worse progression. In contrast, a low level of ERα reduces incidence but promotes tumor evolution to higher grade by inducing cell proliferation and vascularization. Discrete and sparse alterations lead to a non-aggressive phenotype. These data highlight the impact of the ERα expression level on genetic instability, cell growth, and vascularization, therefore explaining the prevalence of high-grade tumors and a predisposition to treatment resistance in men compared to women.

Here, we confirm the importance of estrogen signaling in defining the sex specificity of aggressive lactotroph tumors. While our work demonstrates that a very strong correlation exists between the expression of ERα and the product of its gene *ESR1*, our analysis further reveals that 18% of the genes differentially expressed between male and female lactotroph tumors are involved in estrogen signaling (Table S1). Whereas, a low level of ERα expression correlated with aggressiveness for all tumors from men, out of ten tumors from women, this observation only occurred in the two tumors that were classified as aggressive (tumor grade 2b). The low *ESR1* mRNA level observed in aggressive female lactotroh tumors, may explain the non-significant differential expression of *ESR1* mRNA, between men and women. When the expression data of this gene from the two aggressive women tumors were removed, the differential expression of *ESR1* mRNA became significantly different between men and women lactotroph tumors. We therefore hypothesized that a high level of ERα expression may result in a protective effect against aggressiveness in lactotroph tumors in women. In contrast, the low expression level of ERα found in male tumors may explain the higher risk of more aggressive tumor behavior, recurrence, and resistance to treatment. As the *ESR1* mRNA level was already lower in the normal pituitaries of men compared to women, we suggested that early divergence of the ERα level and sex-associated regulation of estrogen signaling may have a major influence on vascularization, tumor growth, and chromosomic alteration (Figure 3).

Among all the estrogen signaling-associated genes we identified, *STAP2* appears to be one of the most interesting candidates to explore. Indeed, *STAP2* is regulated by estrogen and increases during menopause in women, when the estrogen level decreases (20). Thus, the higher *STAP2* mRNA level in lactotroph tumors from men compared to women may be related to the lower level of ERα. *STAP2* is known to increase cell growth and tumor progression in breast and prostate cancer by interacting with the Brk and STAT pathways (21–23). In male lactotroph tumors, while we found *STAP2* to be increased at all tumor grades, we noted that *STAT3* and *STAT5* were higher in male grade 2b/3 than in female grade 2b tumors (Figure S1). More interestingly, the unphosphorylated form of STAT has been shown to participate in DNA damage protection through the stabilization of the heterochromatin protein 1 (24). Taken together, such observations suggest that the low level of ERα could increase the level of *STAP2* mRNA, therefore contributing to the aggressiveness of male lactotroph tumors through a reduced genome integrity mediated by STAT signaling. Consistent with this hypothesis, we found chromosome abnormalities to be more numerous in both tumors from men and aggressive tumors (14). Even if the following observations as to be confirmed on a larger number of lactotroph tumors, the additional gain of chromosome 19p which was observed only in the 3 aggressive or malignant tumors in man may suggest that specific gains of chromosome 19p could have an influence on tumors aggressiveness. These findings suggest that the sexual dimorphism of lactotroph tumors and their more aggressive behavior in men than in women may be related to increased chromosomic abnormalities in men compared to women.

Besides confirming the importance of estrogen signaling in the sex-related aggressiveness of lactotroph tumors, our work sheds light on the potent role of a series of sex chromosome-related candidates. Indeed, our analysis highlights several X chromosome-located genes that are overexpressed in male lactotroph tumors, especially the cancer-testis antigen (*CTAG2*), the creatine-transporter (*SLC6A8*), and two growth factors (*FGF13* and *VEGFD*). *CTAG2*, normally only expressed in the testis, is involved in the invasive behavior of breast cancer (6) and is highly expressed in gastrointestinal and breast carcinomas (25). Moreover, the cancer-testis antigens are implicated in repressing estrogen signaling (26). In our study, *CTAG2* is specifically upregulated and correlated with markers of aggressiveness *ADAMTS6, AURKB, CCNB1, CENPE*, and *PTTG1* (11, 27) only in male lactotroph tumors. SLC6A8, a transporter that imports extracellular phosphocreatine into the cell inhibited by estrogens, was shown to induce sex differences in creatine metabolism (16). As in lactotroph tumors in men, *FGF13* is upregulated in cancer cells (28, 29). This growth factor is not a cancer driver but serves to enhance survival of cancer cells (28, 29). Indeed, the higher expression of *SLC6A8* and *FGF13* genes may contribute to increased cell survival in lactotroph tumors in men. Not only *VEGFD*, but also an adaptor protein involved in VEGF signaling, *SH2D2A*, and five other genes *LTBP1, ISL1, PTGS1, PTPRZ1*, and *ROBO1* (30–34), known to promote angiogenesis, were overexpressed in men. A high expression of VEGF was observed by immunohistochemistry in 60.7% of the lactotroph tumors (35). Its expression was higher in pituitary carcinomas compared to benign adenomas (36), as was microvascular density (37). One case of our series (case 3) illustrates the importance of VEGF in lactotroph tumors. This malignant lactotroph tumor from a man had abnormalities in chromosome 1, chromosome 11, chromosome 19, a neoangiogenesis (38), and showed endothelial cell expression of endocan, another angiogenic factor which is controlled by VEGF and FGF2 (39). This overexpression of VEGF in lactotroph tumors could be of therapeutic interest. Indeed, it has been also reported that an anti-VEGF (bevacizumab) treatment stabilized the progression of a pituitary carcinoma and induced extensive perivascular fibrosis (40). Finally, despite the fact that lactotroph tumors are less responsive to dopamine agonists in men than in women, we did not find a sex-related difference in the expression of the dopamine agonist receptor D2 itself. However, the growth inhibiting action of dopamine on lactotroph cells is partly mediated by the transforming growth factor (TGF)- β1 system (41). The availability of TGFβ1 is modulated by latent TGFβ-binding proteins (LTBP) and bone morphogenetic protein 1 (BMP1) is one of the activators of latent TGFβ1. Estradiol has been shown to inhibit pituitary LTBP1 expression (42) and we observe in our series a significantly increased LTBP1 expression among males. Sclerostin domain-containing 1 (SOSTDC1), a BMP antagonist, is inhibited by estrogens (43) and appears here to be highly upregulated in males lactotroph tumors. Thus, we can speculate that the lack of ERα observed in men could result in an inhibition of TGFβ1 and thus in an impairment of the control of lactotroph proliferation by dopamine.

This study, based on a combined series of analysis ranging from transcriptomics and CGH array to a detailed literature review, demonstrates that the prevalence of aggressive lactotroph tumors in men is not linked to a single factor. Our data further suggest that among the many factors differentially expressed between lactotroph tumors from men and women, an important subset belongs to the estrogen signaling pathway, while androgen or testosterone signaling molecules are not differently impacted. To that extent, this study enriches our model of lactotroph tumor progression by highlighting that a low expression of ERα is an early factor favoring higher aggressiveness of lactotroph tumor cells in men compared to women. Besides this important observation, our work sheds light on a novel series of mechanisms, identifying a sex-specific gene expression in lactotroph tumors in men that relates to a genetic instability and to the increased expression of several candidate genes promoting angiogenesis, cell proliferation, and survival of lactotroph tumor cells.

## Author contributions

AW, ED, JT, and GR designed research studies. AW conducted experiments and acquired data. AW and ED analyzed data. AW, ED, PB, JL, JT, and GR discussed results. PF, EJ, and PC provided human material and biopsies. AW and ED wrote the manuscript.

### Conflict of interest statement

The authors declare that the research was conducted in the absence of any commercial or financial relationships that could be construed as a potential conflict of interest.

## Supplementary material

Figure S1 STAT3 and STAT5B mRNA expression in aggressive lactotroph tumors. Gray bar were the average relative mRNA expression measured in the 2 aggressive lactotroph tumors in woman classified as 2b. Dark bar were the average relative mRNA expression measured in the 7 aggressive lactotroph tumors in men classified as 2b or 3.

Table S1 Genes differentially deregulated between Men and Women lactotroph tumors.
