# Supplementary figures and images for: Sex-Related Differences in Lactotroph Tumor Aggressiveness Are Associated With a Specific Gene-Expression Signature and Genome Instability
Source: Front Endocrinol (Lausanne). 2018 Nov 30;9:706. doi: 10.3389/fendo.2018.00706 (PMC6283894; doi:10.3389/fendo.2018.00706)

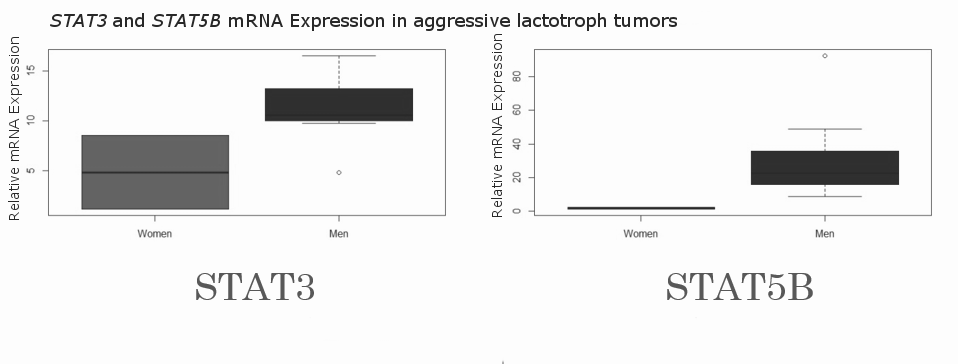

Supplement: Figure S1 — STAT3 and STAT5B mRNA expression in aggressive lactotroph tumors. Gray bar were the average relative mRNA expression measured in the 2 aggressive lactotroph tumors in woman classified as 2b. Dark bar were the average relative mRNA expression measured in the 7 aggressive lactotroph tumors in men classified as 2b or 3. [file Image_1.TIFF]
